# Interleukin-6-knockdown of chimeric antigen receptor-modified T cells significantly reduces IL-6 release from monocytes

**DOI:** 10.1186/s40164-020-00166-2

**Published:** 2020-06-08

**Authors:** Liqing Kang, Xiaowen Tang, Jian Zhang, Minghao Li, Nan Xu, Wei Qi, Jingwen Tan, Xiaoyan Lou, Zhou Yu, Juanjuan Sun, Zhenkun Wang, Haiping Dai, Jia Chen, Guoqing Lin, Depei Wu, Lei Yu

**Affiliations:** 1 Institute of Biomedical Engineering and Technology, Shanghai Engineering Research Center of Molecular Therapeutics and New Drug Development, School of Chemistry and Molecular Engineering, East China Normal University, No, 3663 North Zhongshan Road, Shanghai, 200065 China; 2 Shanghai Unicar-Therapy Bio-medicine Technology Co., Ltd, No 1525 Minqiang Road, Shanghai, 201612 China; 3 National Clinical Research Center for Hematologic Diseases, Jiangsu Institute of Hematology, The First Affiliated Hospital of Soochow University, Suzhou, China; 4 Institute of Blood and Marrow Transplantation, Collaborative Innovation Center of Hematology, Soochow University, Suzhou, China; 5 Central Laboratory of Hematology and Oncology, First Affiliated Hospital, Harbin Medical University, Harbin, 150001 Heilongjiang China; 6 Department of Hematology, Huai’an Hospital Affiliated to Xuzhou Medical College, Huai’an Second People’s Hospital, Huai’an, 223002 China

**Keywords:** CAR-T, CRS, CRES, IL-6, shRNA, safety, B lymphocyte leukemia

## Abstract

**Background:**

T cells expressing a chimeric antigen receptor (CAR) engineered to target CD19 can treat leukemia effectively but also increase the risk of complications such as cytokine release syndrome (CRS) and CAR T cell related encephalopathy (CRES) driven by interleukin-6 (IL-6). Here, we investigated whether IL-6 knockdown in CART-19 cells can reduce IL-6 secretion from monocytes, which may reduce the risk of adverse events.

**Methods:**

Supernatants from cocultures of regular CART-19 cells and B lymphoma cells were added to monocytes in vitro, and the IL-6 levels in monocyte supernatants were measured 24 h later. IL-6 expression was knocked down in regular CART-19 cells by adding a short hairpin RNA (shRNA) (termed ssCART-19) expression cassette specific for IL-6 to the conventional CAR vector. Transduction efficiency and cell proliferation were measured by flow cytometry, and cytotoxicity was measured by evaluating the release of lactate dehydrogenase into the medium. Gene expression was assessed by qRT-PCR and RNA sequencing. A xenograft leukemia mouse model was established by injecting NOD/SCID/γc-/- mice with luciferase-expressing B lymphoma cells, and then the animals were treated with regular CART-19 cells or ssCART-19. Tumor growth was assessed by bioluminescence imaging.

**Results:**

Both recombinant IL-6 and CART-19 derived IL-6 significantly triggered IL-6 release by monocytes. IL-6 knockdown in ssCART-19 cells dramatically reduced IL-6 release from monocytes in vitro stduy. In vivo study further demonstrated that the mice bearing Raji cells treated with ssCART-19 cells showed significant lower IL-6 levels in serum than those treated with regular CART-19 cells, but comparable anti-tumor efficacy between the animal groups.

**Conclusion:**

CAR T-derived IL-6 is one of the most important initiators to amplify release of IL-6 from monocytes that further drive sCRS development. IL-6 knockdown in ssCART-19 cells by shRNA technology provide a promising strategy to improve the safety of CAR T cell therapy.

## Background

T cells expressing a chimeric antigen receptor (CAR) engineered to target CD19 are an effective treatment for refractory and relapsed acute B lymphoblastic leukemia [1]. However, CAR T cell therapy is also associated with severe life-threatening side effects [2–5], which have greatly hindered its implementation. As many as 70% of patients on this therapy experience severe cytokine release syndrome (sCRS) [4], and 40% of patients experience severe CAR T cell-related encephalopathy (sCRES) [1, 6, 7]. Both of these complications are associated with high IL-6 levels in the circulation and cerebrospinal fluid [2]. While tocilizumab and hormone therapy can be used to treat sCRS [8], these approaches are costly and increase the risk of additional side effects such as infection, and more importantly, monoclonal antibodies such as tocilizumab cannot reach damaged areas in the brain because of the brain–blood barrier [9]. Hormone therapy can also impair CAR T cell function and weaken therapeutic efficacy [10, 11]. A novel and effective method to improve the safety of CAR T cell clinical application without affecting the efficacy of CAR T cells is urgently needed.

After adoptive transfer into patients, CAR T cells are activated by B cells to proliferate and release various proinflammatory cytokines, including interleukin (IL)-6, interferon (IFN)-γ, MCP-1, and granulocyte macrophage colony-stimulating factor (GM-CSF) [12–14]. This activation leads to target cell lysis and widespread immune activation [12, 15]. At the same time, the cytokines produced by the CAR T cells or other immune cells, such as monocytes, in response to CAR T cell infusion appear to drive CRS and CRES [12, 15].

Among these cytokines, IL-6 appears to be a key driver of sCRS, sCRES and related death [16]. One study reported that these syndromes are triggered by CAR T cell activation and proliferation after recognition of CD19+ target cells and are characterized by elevated serum levels of IL-6 and IFN-γ [12, 15]. Elevated IL-6 expression has also been associated with neurotoxicity caused by CAR T cells [17]. While CAR T cells themselves produce IL-6 after infusion, other cells, including monocytes, dendritic cells, B lymphocytes and endothelial cells, may contribute to IL-6 release [3, 18–21]. High levels of cytokines such as IL-6 and IFN-γ may activate endothelial cells, resulting in the release of Von Willebrand Factor (VWF) and angiopoietin-2, which in turn amplify endothelial activation [22]. The mechanisms that trigger the rapid release of large amounts of IL-6 from monocytes and endothelial cells during CAR T therapy are unclear.

Given the important role of IL-6 in complications related to CAR T cell therapy, we first investigated whether CAR T cell-derived IL-6 activates and triggers monocyte release of proinflammatory cytokines, including IL-6, and then examined whether IL-6 production in monocytes can be reduced by knocking down IL-6 gene expression in CAR T cells without affecting their antitumor efficacy. The current study will provide useful data for the development of a new strategy to improve the safety of CAR T cell therapy.

## Materials and methods

### Generation of IL-6 shRNA-expressing CAR constructs

We designed 8 different short hairpin RNA (shRNA) sequences targeting the 3′ untranslated region (UTR) of the human IL-6 gene using human U6 as the promotor (sequence: 5′AATTCAAAAAAGGGCACAGAACTTATGTTGTTCTCGAGAGAACAACATAAGTTCTGTGC-3′). Different shRNAs were synthesized by GENERAY Biotech (Shanghai, China) and inserted into a CAR construct (Unicar-Therapy Bio-medicine Technology Co., Ltd., Shanghai, China) containing a CD19-targeted single-chain variable fragment (FMC63), the EF1a promoter, the costimulatory 4-1BB domain, and the CD3 zeta domain. The construct was cotransfected with three packaging plasmids into HEK 293T packaging cells, and the resulting lentiviruses were isolated, concentrated by ultracentrifugation, and immediately stored at − 80 °C.

### CART-19 cells and target cell lines

Peripheral blood mononuclear cells (PBMCs) were obtained from healthy donors by gradient centrifugation using Lymphoprep™ (Oriental Hua Hui, Beijing, China). For CAR T cell preparation, T lymphocytes were purified using anti-CD3 positive-selection beads (Miltenyi Biotec, Bergisch-Gladbach, Germany) and stimulated with anti-CD3/CD28 monoclonal antibodies (Miltenyi Biotec) for 18–24 h. The T cells were then transduced with recombinant lentiviral vectors for 48 h. The CAR T cells were cultured for 12–14 days in AIM-V medium (Gibco, NY, USA) supplemented with 10% autologous human serum, 100 IU/mL recombinant human IL-2 (PeproTech, Rocky Hill, USA), 5 ng/mL recombinant human IL-7 (PeproTech) and 5 ng/mL recombinant human IL-15 (PeproTech).

Monocytes were collected as previously described [23]. In total, 2 × 10^6^ cells were seeded per well in 1 mL of RPMI 1640 medium (Gibco) supplemented with 0.1 mmol/L minimum essential medium containing nonessential amino acids (Solarbio, Beijing, China), 2 mmol/L l-glutamine (Thermo Fisher, Waltham, USA), and 10% fetal bovine serum (FBS; Gibco). Cells were supplemented with fresh medium every 2 days, harvested using 2 mmol/L ethylenediaminetetraacetic acid (EDTA, Klamar, Shanghai, China) and stained with an APC-conjugated antibody against CD14 (BD Bioscience, New Jersey, USA) to confirm differentiation.

A CD19-expressing Burkitt human B lymphoma (Raji) cell line and the K562 human leukemia cell line (ATCC, Manassas, VA, USA) were used as target cells in vitro and as control cells. The cells were maintained in RPMI 1640 medium (Gibco) supplemented with 10% FBS (HyClone, UT, USA) according to manufacturer protocols. Raji cells were transfected with a firefly luciferase construct (Unicar-Therapy Bio-medicine Technology Co., Ltd., Shanghai, China) using a lentiviral vector, and stably transfected cells were selected using puromycin.

### Coculture assay

An “effector/target cell” culture model was used for the coculture assay. CAR T cells (effector cells) and Raji cells (target cells) were incubated at a ratio of 5:1 for 24 h in 100 µL of T cell culture medium AIM-V (Gibco) in 96-well plates. The culture supernatant was then added to primary monocytes, and IL-6 levels in the supernatant were measured using the Th1/Th2 Cytometric Bead Array Kit II (BD Bioscience). The IL-6 levels at 0 h (immediately after the addition of the coculture supernatant) were used as the baseline.

Then, 10 ng/mL human recombinant IL-6, IL-2, IL-1 or IFN-γ (PeproTech) was added to the monocyte cultures. Supernatant samples were collected at 6 h, 24 h and 48 h after beginning the coincubations of the different cytokines and monocytes, and monocytes incubated with an equal volume of saline were used as a control for comparisons of cytokine profiles in this experiment.

For re-stimulation cocultures, CAR T cells expressing an shRNA against IL-6 (ssCART-19) or regular CAR T cells were cocultured with Raji cells at a ratio of 5:1 for 6 days, and the different types of CAR T cells were re-stimulated with Raji cells every 36 h. Supernatants were collected every 12 h, and the levels of IL-6, IFN-γ and IL-2 were measured with the Th1/Th2 Cytometric Bead Array Kit II.

### Flow cytometry

All samples were washed twice in 0.1 mL of phosphate-buffered saline (PBS) containing 2% FBS. The transduction efficiency and CD4/CD8 ratio were determined by labeling CAR T cells with a FITC-labeled human CD19 protein, Fc Tag (CD9-HF251, ARCO, Biosystems, Beijing, China), an APC-conjugated antibody against CD8 (catalog no. 344722, BioLegend, California, USA), and a PE-Cy7-conjugated antibody against CD4 (25-0047-42, eBioscience, San Diego, CA) at 4 °C for 45 min in the dark.

For assessing the CAR T cell phenotype to analyze CAR T cell differentiation stages, CAR T cells were incubated with the following antibodies: APC-Cy7-conjugated anti-CD8 (344714, BioLegend), AF700-conjugated anti-CD4 (56004942, eBioscience), PE-conjugated anti-CCR7 (353204, BioLegend), PerCP-Cy5.5-conjugated anti-CD45RA (304122, BioLegend), and PE-Cy7-conjugated anti-CD127 (25-1287-42, eBioscience).

CD19 expression on Raji cells was detected by staining with an APC-conjugated antibody against CD19 (302212, BioLegend), and monocyte CD14 expression was determined by staining with an Alexa Fluor 700-conjugated antibody against CD14 (325614, BioLegend).

After antibody labeling, samples were washed twice in 0.1 mL of PBS containing 2% FBS before detection using an Attune NxT flow cytometer (Thermo Scientific, USA). Data were analyzed using FlowJo V10 (TreeStar, USA).

### Cell proliferation assay

The cell proliferation assay was performed using a carboxyfluorescein diacetate succinimidyl ester (CFSE) assay kit (Abcam, UK) following the manufacturer’s instructions. In brief, 2 × 10^5^ CART-19 cells were labeled with 2.5 μM CFSE and cocultured with Raji cells, which were inactivated by exposure to 10 µg/mL mitomycin C (Selleck, Houston, USA) at a ratio of 2:1 in 24-well plates in 200 μL of serum-free AIM-V medium (Gibco, USA) for 5 days. CFSE was detected by flow cytometry on days 0 and 5 to analyze CAR T cell proliferation.

### Degranulation assay

CD107a expression on CD8 T cells was detected by flow cytometry. In brief, 1 × 10^6^ CAR T cells were cocultured with target cells at a 5:1 ratio in 96-well plates with a total of 200 µL of T cell culture medium AIM-V (Gibco) per well, The Golgi inhibitor Monessen (Invitrogen, Carlsbad, US) and CD107a-AF700 antibody (BD, New Jersey, USA) were added before coincubation. A protease inhibitor cocktail (Invitrogen, Carlsbad, US) was added to the well containing the positive control. After 6 h incubation, the cells were collected and washed twice before analyzed by flow cytometry.

### Cytotoxicity assay

Raji cells (1 × 10^4^) and CART-19 cells were seeded in 200 µL of serum-free RPMI 1640 medium (Gibco) at an effector: target (E:T) ratio of 10:1, 5:1, 2.5:1, or 1.25:1 in 96-well plates for 16 h. Lactate dehydrogenase (LDH) activity in the medium was measured using a cytotoxicity detection kit (Promega, Wisconsin, USA) according to the manufacturer’s protocol, and the absorption at 490 nm was measured using a full wavelength reader Multiskan GO (Thermo Scientific). The percentage of tumor lysis was calculated as follows: % tumor lysis = (experimental value − low control of CART-19 cells − low control of target cells) × 100/(high control of target cells − low control of target cells), where the low control was the assay medium + cells and the high control was the assay medium + 2% Triton X-100 + cells.

### Quantitation of cytokine levels

Cytokine levels in culture supernatants were measured using the Th1/Th2 Cytometric Bead Array Kit II (BD Bioscience) according to manufacturer protocols. In brief, supernatants collected from the coculture system or mouse serum was incubated with fluorophore-labeled antibodies against IL-2, IL-4, IL-6, IL-10, IFN-γ, TNF-α and IL-17A for 3 h. After the incubation, the samples were washed with the wash solution and analyzed by flow cytometry.

### Quantitative RT-PCR

Total mRNA was extracted from cells using TRIzol (Invitrogen) and converted into cDNA using HiScript^®^ II Q RT SuperMix (Vazyme Biotech, Nanjing, China). Quantitative RT-PCR was performed using an ABI-7500 system (Life Technologies, USA). The primer sequences used for RT-PCR are as follows: IL-6, forward: 5′-TAACCACCCCTGACCCAACCA-3′, reverse: 5′-GCGCAGAATGAGATGAGTTGTCA-3′, a fluorescent probe, 5′-AAATGCCAGCCTGCTGACGAAGCTGCA-3′; glyceraldehyde-3-phosphate dehydrogenase (GAPDH), forward:5′-GGACAGGACCATATTGAGGGACA, reverse: 5′- AGGAGTGAGTGGAAGACAGAATGGA, a fluorescent probe, TGGAAGGAGCACTTCATCTGTT; IL-1α, forward: 5′-GCGTTTGAGTCAGCAAAGAAGTC, reverse: 5′-GGAGTGGGCCATAGCTTACA; and IL-1β, forward: 5′-TCGCCAGTGAAATGATGGCT, reverse: 5′-TGGAAGGAGCACTTCATCTGTT. Expression relative to that of the control sample was calculated as follows: 2 −ΔΔCt = 2 ^−(ΔCt [sample] − ΔCt [control]).

### RNA extraction and purification

Total RNA was extracted using TRIZOL Reagent (Cat#15596-018, Life technologies, Carlsbad, CA, US) following the manufacturer’s instructions and checked for a RIN number to inspect RNA integrity by an Agilent Bioanalyzer 2100 (Agilent technologies, Santa Clara, CA, US). Qualified total RNA was further purified by RNA Clean XP Kit (Cat A63987, Beckman Coulter, Inc. Kraemer Boulevard Brea, CA, USA) and RNase-Free DNase Set (Cat#79254, QIAGEN, GmBH, Germany).

### Xenograft model

All experiments were approved by the Institutional Animal Care and Use Committee of East China Normal University. Male NOD/SCID/γc-/- (NSG) mice aged 4 to 6 weeks were obtained from Biocytogen (Beijing, China). Mice were injected with 1 × 10^6^ Raji cells that stably expressed luciferase (Raji-luc) via the tail vein to establish a xenograft leukemia tumor model. The mice were then randomly divided into four groups and treated with saline (n = 3, control), 6 × 10^6^ untransduced T cells (n = 3, negative treatment control), 6 × 10^6^ regular CART-19 cells (n = 3), or 6 × 10^6^ ssCART-19 cells (n = 3) on day 4. Tumor progression was monitored by bioluminescence imaging (BLI) using a Xenogeny-IVIS imaging system (Perkin Elmer, MA, USA) every 3 days beginning on day 1. The mice were sacrificed by vertebral dislocation when moribund or upon development of hind-limb paralysis.

For in vivo imaging of Raji-luc cells, mice were injected intraperitoneally with D-luciferin (YEASEN, Shanghai, China), anesthetized with isoflurane and imaged using the Xenogeny-IVIS imaging system (Perkin Elmer).

### Statistical analysis

Statistical analyses were carried out using Prism 8.0 (GraphPad Software Inc., San Diego, CA, USA). Each experiment was repeated with three biological replicates, and data are presented as the mean ± SD. The group means from degranulation assays and cytokine secretion assays were compared using one-way analysis of variance (ANOVA). Differences among treatment groups and control groups in vitro killing assays were analyzed using two-way ANOVA followed by Dunnett’s multiple-comparison test. Differences associated with a *p*-value < 0.05 were considered significant.

## Results

### IL-6 derived from activated regular CART-19 cells triggers monocyte release of IL-6

**Fig. 1:**
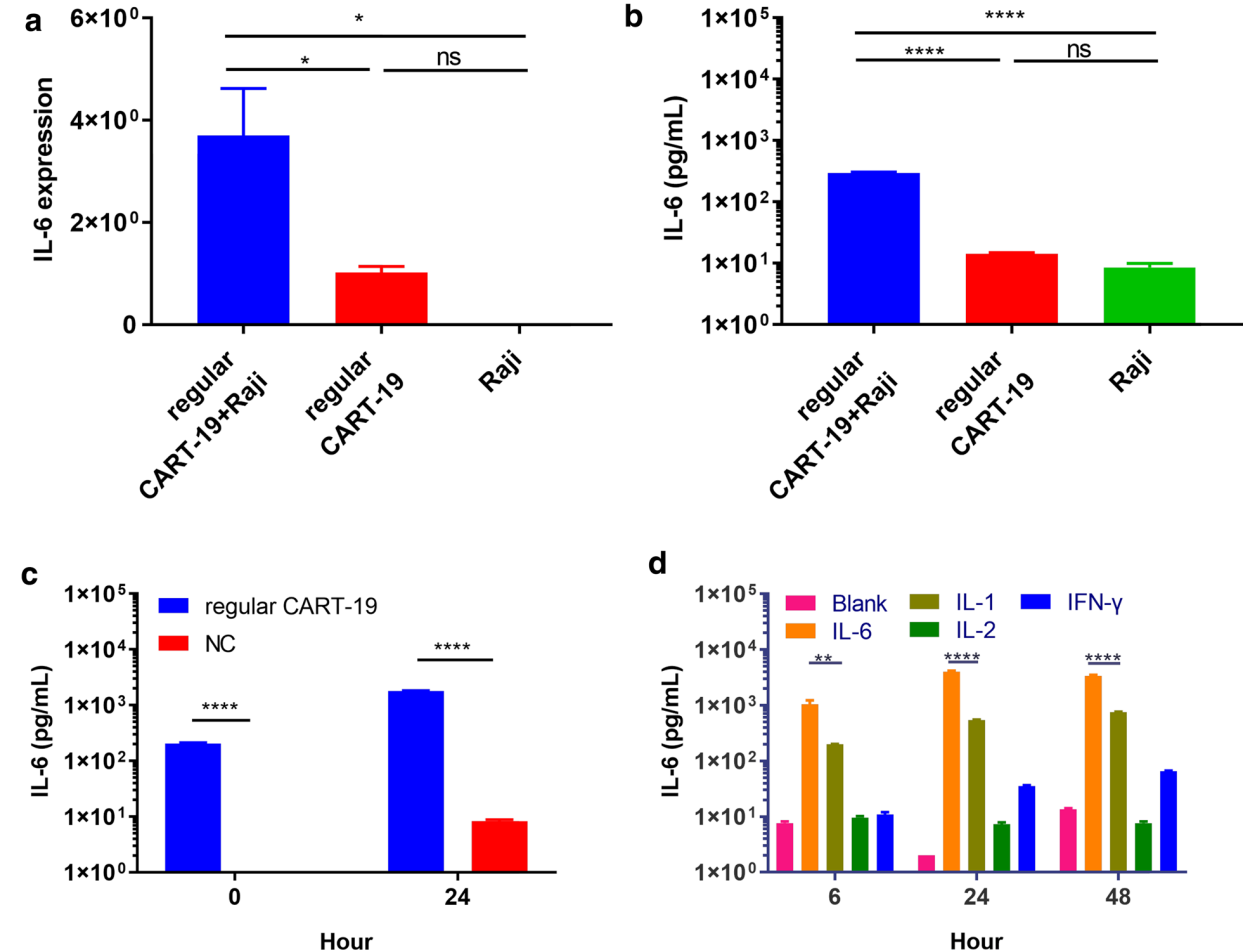
IL-6 derived from activated CART-19 cells triggers monocyte release of IL-6. **a** IL-6 mRNA levels in CART-19 cells measured by quantitative PCR. Regular CART-19 cocultured with Raji cells for 24 h, IL-6 mRNA levels were measured by quantitative PCR. Data are shown as the mean ± SD (n = 3). NS = no significant difference, *p < 0.05. **b** IL-6 cytokines level secreted into the supernatant. IL-6 protein levels in the supernatants of in CART-19 cells cocultured with Raji cells for 24 h, were measured using the Th1/Th2 Cytometric Bead Array Kit II. NS, no significant difference, ****p < 0.0001. **c** IL-6 protein levels in the supernatants of cultures of monocytes incubated with supernatants from CART-19/Raji cell cocultures, as measured using the Th1/Th2 Cytometric Bead Array Kit II. NC, untransduced T cells cocultured with Raji cells. **d** IL-6 protein levels in monocyte culture medium after the addition of recombinant IL-1, IL-2, IL-6 or IFN-γ. Data are shown as the mean ± SD (n = 3). Student’s *t* test was used to assess differences between groups. **p ≤ 0.01, ****p ≤ 0.0001

To determine the effect of cytokines from activated regular CART-19 cells on monocytes, we first examined whether regular CART-19 cells increase the expression of IL-6 and other cytokines upon encountering CD19+ cells. We cocultured regular CART-19 cells with CD19-expressing Burkitt human B lymphoma (Raji) cells in an “effector/target” coculture model system. CD19 expression on Raji cells was confirmed by FACS (Additional file 1: Figure S1a). Significant increases in the amounts of both IL-6 mRNA production and IL-6 cytokine production were observed in the cocultured regular CART-19 cells compared with nonactivated regular CART-19 cells, with the increases being four fold and 20-fold higher, respectively (Fig. 1a, b), which demonstrated that regular CART-19 cells could significantly increase IL-6 mRNA and protein expression once induced specifically by Raji cells.

We next examined whether the enhanced IL-6 production by activated regular CART-19 cells triggers monocytes to increase the release of IL-6 and other cytokines by adding supernatants from CAR T/Raji cell cocultures to CD14-expressing monocytes (see Additional file 1: Figure S1b for confirmation of CD14 expression). Significantly higher IL-6 levels in the monocyte culture medium were observed after 24 h of incubation with the supernatant of the “effector/target” coculture than immediately after beginning the incubation, which was used as the baseline level for comparison (Fig. 1c). In contrast, there were no differences in IL-6 or other cytokine levels in the monocyte culture medium after the addition of supernatants from untransduced T cells cocultured with Raji cells when comparing the same study time points. To pinpoint which cytokines are the main triggering factors that enhance IL-6 release from monocytes, we incubated primary monocytes with recombinant IL-1, IL-2, IL-6, or IFN-γ for 48 h, and then the released IL-6 level was measured by flow cytometry. To determine whether the enhancement in the IL-6 level is derived from activated monocytes, the amount of exogenous IL-6 was subtracted from the level measured in the monocyte culture medium. Incubation with IL-6 triggered a remarkable enhancement in the release of IL-6 from monocytes, with almost 100 times more IL-6 release in the IL-6-treated group than in the negative control group that was not incubated with any cytokines (Fig. 1d). The increases in IL-6 levels were also observed to be 25 and 3 times higher for the monocytes incubated with IL-1 or IFN-γ than for the negative control monocytes, respectively. In contrast, IL-2 did not trigger IL-6 release. These data demonstrated that IL-6 was the most potent cytokine in triggering monocyte IL-6 release under our experimental conditions.

### IL-6 knockdown does not impair basic properties of the ssCART-19 T cells

**Fig. 2:**
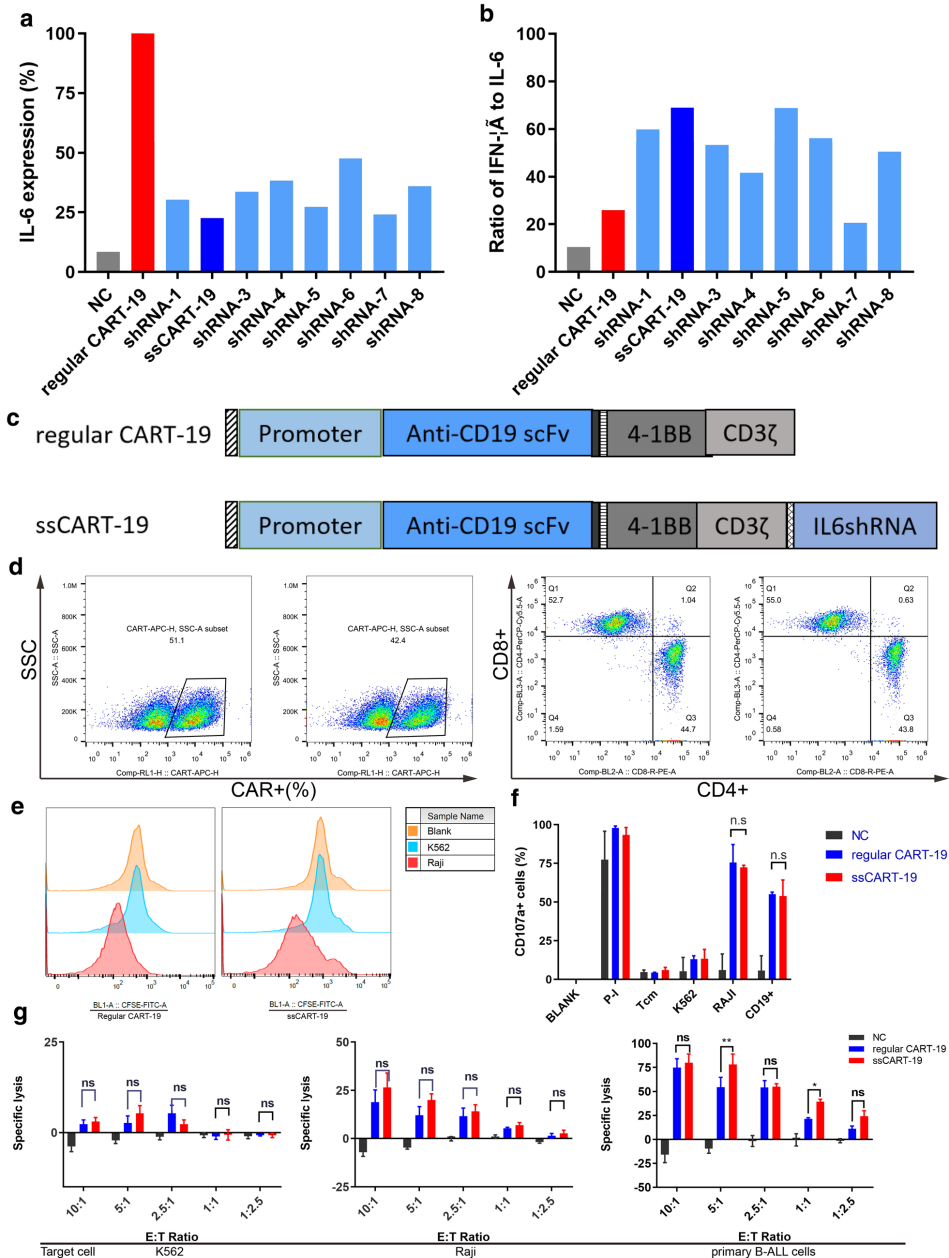
IL-6 knockdown in ssCART-19 T cells does not impair basic properties of CAR T cells. **a** IL-6 mRNA levels and **b** IFN-γ/IL-6 mRNA ratios in CART-19 cells expressing 8 different IL-6-specific shRNAs. **c** Schematic of the CD19 CAR vector containing an anti-human CD19 scFv linked to 4-1BB costimulatory domains and CD3-ζ signaling domain with (ssCART-19) or without (regular CART-19) an IL-6-specific shRNA modification. **d** Transduction efficiency and the CD4/CD8 ratio of ssCART-19 and CART-19 cells. **e** Cell proliferation of ssCART-19 cells and regular CART-19 cells after re-stimulation with Raji cells, as analyzed by flow cytometry. **f** CD107a expression in ssCART-19 cells and regular CART-19 cells after induction with Raji cells. **g** Cytotoxicity of ssCART-19 and CART-19 cells to K562 cells, Raji cells and autologous primary acute B lymphocytic leukemia cells at different effector: target ratios. Differences among groups were assessed for significance by using one-way ANOVA. Data are shown as the mean ± SD (n = 3). NS, no significant difference, *p ≤ 0.05, **p ≤ 0.01; ***p ≤ 0.001, ****p ≤ 0.0001

To investigate whether the introduction of an IL-6-specific shRNA to regular CART-19 cells changes basic cell properties, we first designed 8 different IL-6-targeting shRNA sequences and cloned them into lentiviral vectors containing the CAR construct. IL-6 shRNA-2, which had the best IL-6 gene knockdown efficiency (70%) (Fig. 2a) and the highest IFN-γ/IL-6 mRNA ratio (73%) (Fig. 2b), was selected for this study. We then constructed CAR vectors containing a 4-1BB costimulatory domain, CD3 zeta domain and CD19-targeted single-chain variable fragment (FMC63) with or without IL-6 shRNA-2 (Fig. 2c). T cells transduced with CD19-CAR were called regular CART-19 cells, and those transduced with CD19-CAR and IL-6 shRNA-2 were designated ssCART-19 cells for subsequent experiments.

Then, the basic properties of ssCART-19 cells, including transduction efficiency, the CD4/CD8 ratio after transduction, proliferation, and cytotoxicity to the corresponding cancer cells were characterized and compared with the same features of regular CART-19 cells. The ssCART-19 cells showed cell properties similar to those of the regular CAR T-19 cells in terms of the level of transduction efficiency (45.7% vs 38.1%), the ratio of CD4/CD8 (Fig. 2d), proliferation (Fig. 2e) and cytotoxicity as measured by a CD107a (Fig. 2f) degranulation assay or cytotoxicity assay when cocultured with either Raji cells or patient-derived primary B-ALL cells (Fig. 2g). Thus, the results presented here indicated that compared with the regular CART-19 cells without the IL-6-specific shRNA modification, the ssCART-19 cells modified with the IL-6-specific shRNA did not show significant differences from basic T cell functions.

### IL-6 knockdown in ssCART-19 cells reduces IL-6 release from monocytes

**Fig. 3:**
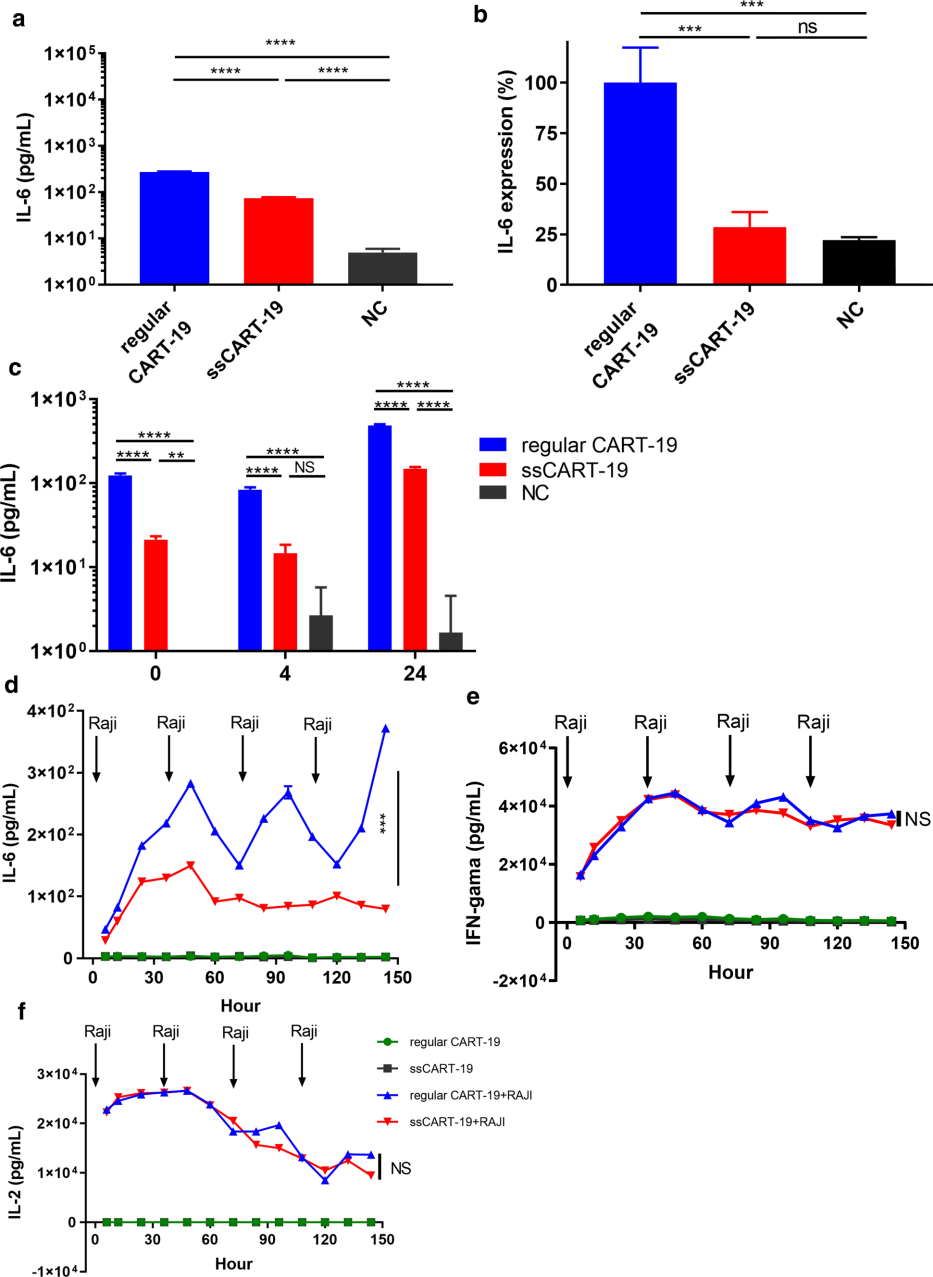
IL-6 knockdown in ssCART-19 T cells reduces IL-6 release from monocytes. **a** IL-6 mRNA levels and **b** IL-6 protein levels in ssCART-19 cells and regular CART-19 cells after stimulation with Raji. Differences were assessed for significance using one-way ANOVA. Data are expressed as the mean + SD. NS, no significant difference, ***p < 0.001. **c** IL-6 levels in the medium of primary monocyte cultures incubated with supernatants from regular CART-19 or ssCART-19 cells cocultured with Raji cells. Differences were assessed using one-way ANOVA. NS, no significant difference. **p ≤ 0.01; ***p ≤ 0.001, ****p ≤ 0.0001. **d** IL-6, **e** IFN-γ and **f** IL-2 protein levels in the supernatants of ssCART-19 or regular CART-19 cells cocultured with Raji cells. The cells were re-stimulated every 36 h as indicated by the black arrows. Differences were assessed for significance using a paired T test. ***p < 0.001

To investigate whether monocyte release of IL-6 can be reduced by knocking down IL-6 expression in ssCART-19 cells, we first confirmed that shRNA-mediated IL-6 knockdown in ssCART-19 cells could significantly reduce soluble IL-6 protein levels to fourfold lower than the levels in regular CART-19 cells (Fig. 3a). Next, we found that IL-6 mRNA levels were reduced by up to 70% in ssCART-19 cells compared to regular CART-19 cells (Fig. 3b). In addition, we also observed that compared with NC T cells, both the regular CART-19 and ssCART-19 cells increased expression and secretion of IL-1α (Additional file 2: Figure S2A&B) but not IL-1β (Additional file 2: Figure S2C&D) when co-cultured with Raji cells. However, there were no significant differences between the regular CART-19 and ssCART-19 cells both at mRNA and protein levels of IL-1α (Additional file 2: Figure S2A&B).

We investigated the effect of IL-6 knockdown in ssCART-19 cells on monocyte IL-6 secretion by adding the supernatant from a coculture of ssCART-19 cells and Raji cells to monocytes. Significantly lower IL-6 levels were observed in the monocyte supernatant after the addition of the supernatant from ssCART-19/Raji cell cocultures than after the addition of the supernatant from regular CART-19/Raji cell cocultures (Fig. 3c). These results indicated that IL-6 knockdown in ssCART-19 cells could remarkably reduce IL-6 secretion by monocytes.

To confirm that IL-6-specific shRNA knockdown activity is stable, specific and continuous, we repeatedly stimulated ssCART-19 cells with Raji cells every 36 h and continuously monitored IL-6 levels in the supernatant. The ssCART-19 cells consistently maintained lower IL-6 levels than regular CART-19 cells over 6 days, regardless of whether they were re-stimulated with Raji cells (Fig. 3d), while the IL-6 expression level of the regular CART-19 cells was significantly enhanced upon re-stimulation with Raji cells. Thus, IL-6-specific shRNA-mediated knockdown could be continuously maintained in ssCART-19 cells without being affected by repeated stimulation with target cells during the experimental period.

### IL-6 knockdown in ssCART-19 cells does not show significant off-target effects

To determine whether IL-6 knockdown affects ssCART-19 cell function, we measured IFN-γ and IL-2 release in ssCART-19 cells. Without Raji cell stimulation, the levels of IFN-γ and IL-2 release from both regular CART-19 and ssCART-19 cells were maintained at baseline levels for the 6 days of the experiment. When activated with Raji cells, the increases in the levels of IFN-γ (Fig. 3e) and IL-2 (Fig. 3f) for the ssCART-19 and regular CART-19 cells were observed to have similar enhancement profiles, which indicated that IL-6 knockdown did not affect IFN-γ or IL-2 expression in CART-19 cells.

We also investigated whether IL-6 knockdown affects the differentiation of ssCART-19 cells by measuring the Tscm, Tcm, Tem and Teff ratios (indicative of different differentiation stages) of populations of CD8+ ssCART-19 cells in comparison to those of population of regular CART-19 cells derived from the same healthy donor. Similar differentiation profiles were observed in both CD8+ T cells for ssCART-19 and regular CART-19 cells (Additional file 3: Figure S3A), which indicated that the modification with the IL-6-specific shRNA knockdown system did not affect the differentiation of ssCART-19 cells.

To determine whether IL-6 knockdown affects the expression of other genes in cells, RNA sequencing (RNA-seq) analysis was conducted. The mRNAs of both regular CART-19 cells and ssCART-19 cells were analyzed by RNA-seq. Compared with the gene expression profiles of both ssCART-19 and regular CART-19 cells, those of ssCART-19 cells showed altered Jak-STAT signaling pathway (CISH), which is involved in IL-6 production (Additional file 3: Figure S3B). Additionally, gene ontology enrichment analysis showed that IL-6 knockdown did not affect T cell activation, cell proliferation or cell differentiation (Additional file 3: Figure S3C), consistent with the results of our in vitro assays. The RNA-seq data indicated that IL-6-specific shRNA knockdown did not obviously produce “off-target effects” that were disease related or T cell function related.

### IL-6 knockdown in ssCART-19 cells improves in vivo safety and preserves antitumor efficacy

**Fig. 4:**
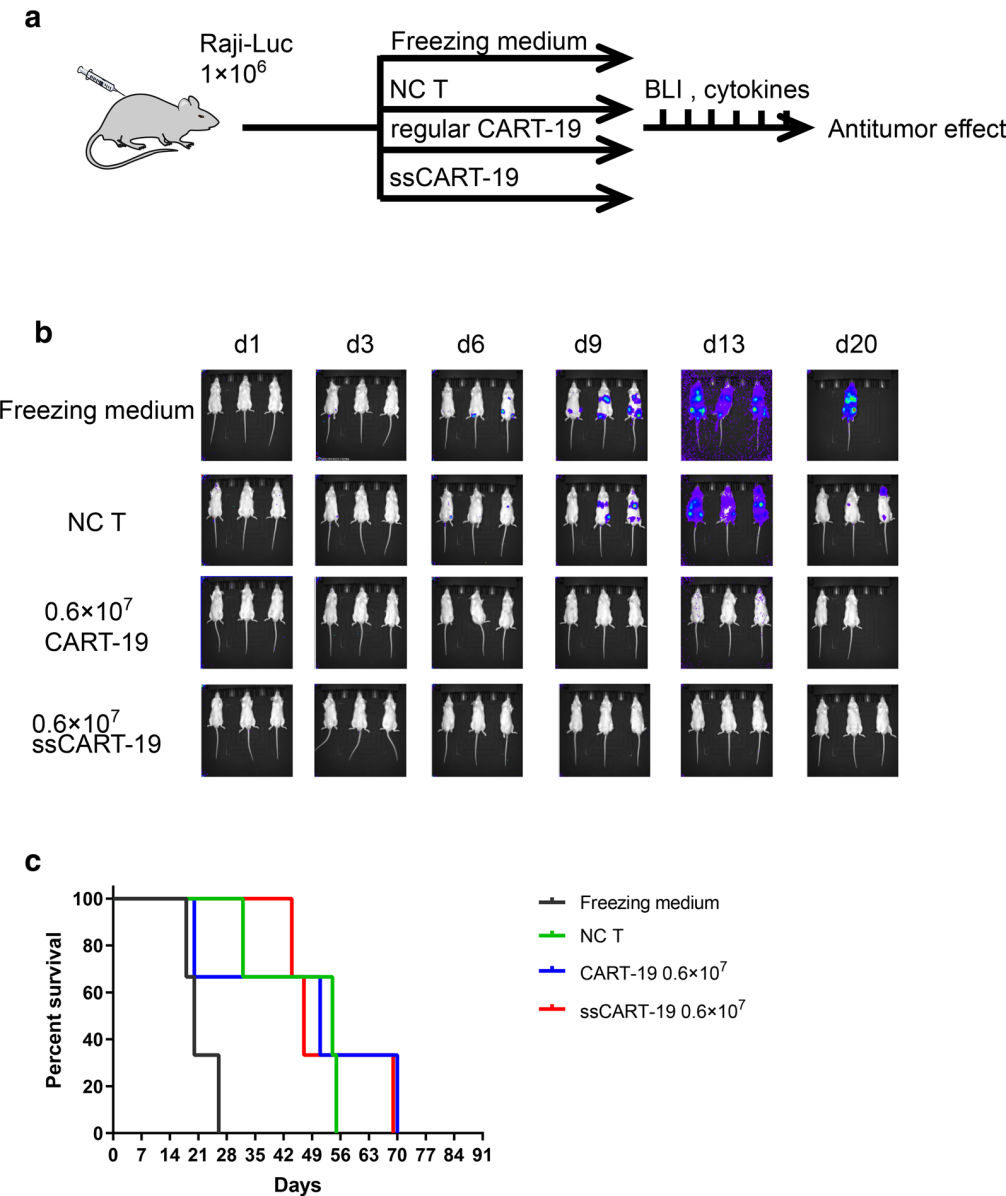
IL-6 knockdown in ssCART-19 cells preserves antitumor efficacy in vivo. **a** Schematic of the Raji-luc xenograft model. NOD/SCID/γc-/- (NSG) mice were injected via the tail vein with 1 × 10^6^ Raji-luc cells on day 0. Bioluminescence imaging (BLI) was performed on day 1 to quantify engraftment. The mice were then randomized and treated with freezing medium (n = 3), untransduced T cells (n = 3), 6 × 10^6^ regular CART-19 cells (n = 3), or 6 × 10^6^ ssCART-19 cells (n = 3). **b** Images showing BLI of tumor growth in mice. Mice treated with both ssCART-19 cells and regular CART-19 cells showed obviously tumor inhibition, however, rapidly tumor progression were observed in the mice treated untransduced T cells, or freezing medium. **c** Survival of mice treated with regular CART-19 cells, ssCART-19 cells, untransduced T cells, or freezing medium

**Fig. 5:**
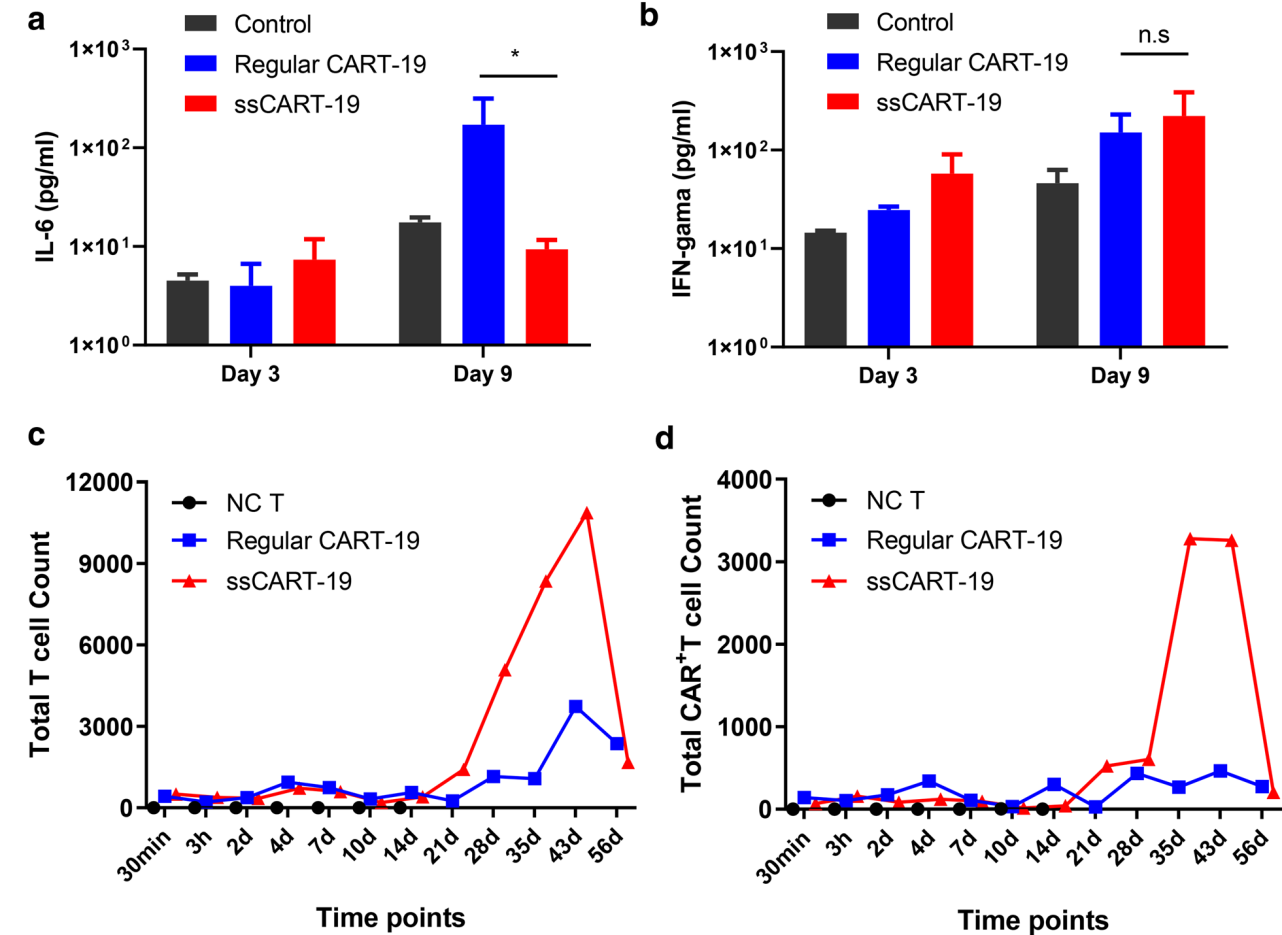
IL-6 knockdown in ssCART-19 cells reduce the IL-6 release in vivo. **a** Serum levels of IL-6 and **b** IFN-γ in the peripheral blood of mice treated with regular CART-19 cells or ssCART-19 cells. Blood was taken on day 3 (1 day before CART-19 cell administration) and day 9 (5 days after CART-19 cell administration). **c** Total T cells count and **d** Total CAR + T cells count in the peripheral blood of mice treated with regular CART-19 cells, ssCART-19 cells, untransduced T cells, or freezing medium measured by flow cytometry

We next assessed the antileukemic activity of ssCART-19 cells in vivo using a xenograft mouse model (see schematic in Fig. 4a) established by injecting NSG mice with 1 × 10^6^ Raji-luc cells on day 0; mice were treated by intravenous infusion of equal numbers of ssCART-19 or regular CART-19 cells (6 × 10^6^) 4 days later. Tumor progression was monitored by BLI every 3 days beginning on day 1. Control mice in the negative control groups were treated with untransduced (NC) T cells or freezing medium and showed rapid progression of leukemia. In contrast, the mice treated with either regular CART-19 or ssCART-19 cells showed obvious inhibition of tumor progression compared to the control mice (Fig. 4b) and increased survival rates (Fig. 4c), and there was no significant difference between the ssCART-19 and regular CART-19 cells, which indicated that ssCART-19 cells had therapeutic efficacies equivalent to those of regular CART-19 cells. As expected, IL-6 levels were significantly lower in the serum of the ssCART-19 cell-treated mice than in that of the regular CART-19 cell-treated mice (Fig. 5a). Importantly, the serum IFN-γ levels in the ssCART-19 cell-treated mice were comparable to those in the regular CART-19 cell-treated mice (Fig. 5b). The mice treated with ssCART-19 cells showed slightly increase in expansion abilities in both total T cells and CAR T cells than those treated with regular CART-19 cells (Fig. 5c, d). Our results demonstrated that compared to regular CART-19 cells, ssCART-19 cells could significantly reduce the IL-6 level in treated mice, but importantly, ssCART-19 cells also showed remarkable antitumor efficacy with IFN-γ release profiles similar to those of regular CART-19 cells under the experimental conditions.

## Discussion

Although CAR T cell therapy has shown remarkable success in the treatment of refractory and relapsed hematological malignancies, safety is a major concern in the clinic due to the life-threatening side effects of severe cytokine release syndrome (sCRS) and severe CAR-T-related encephalopathy (sCRES) [24, 25]. Both sCRS and sCRES are commonly considered to be caused by rapidly elevated levels of proinflammatory cytokines, especially IL-6, after CAR T cell activation by the corresponding target expression on target cells [2]. These proinflammatory cytokines are reportedly expressed by many immune cells, including T cells, B cells, monocytes (dendritic cells and macrophages), and endothelial cells [12, 15].

In this study, we showed that IL-6 secreted by activated regular CART-19 cells triggered the release of relatively large amounts of IL-6 and other cytokines from monocytes. This cascade may be the primary mechanism underlying CRS during CAR T cell therapy. We modified CAR T cells with an IL-6-specific shRNA system to reduce their IL-6 expression. An in vitro study demonstrated that IL-6 knockdown by the IL-6-specific shRNA in ssCART-19 cells significantly reduced ssCART-19 cells IL-6 expression, which in turn reduced the stimulatory activity that induced monocytes to release IL-6. Moreover, the IL-6 knockdown in ssCART-19 cells was maintained during repeated stimulation with B lymphoma cells for 6 days in the study setting, indicating that the IL-6-specific shRNA could act as a long-term suppressor of IL-6 in CAR T cell therapy.

On the other hand, IL-1 has been reported to be a main proinflammatory cytokine in inducing monocyte IL-6 release [6]. However, in our in vitro study, the addition of IL-6 to monocyte culture produced more potent induction of monocyte release of IL-6 and proinflammatory cytokines than did IL-1, which suggests that CART-derived IL-6 is more potent initiator than IL-1 for triggering monocyte IL-6 release and promoting CRS development in patients treated with CAR T cell therapy. In addition, The results showed that the IL-1α expressed from regular CART-19 and ssCART-19 cells were increased at similar levels with both protein and mRNA analysis, but only very low levels of IL-1β were detected from both regular CART-19 and ssCART-19 cells and showed almost as low as negative control T cells’, which indicates that inhibition of IL-6 expression by shRNA technology in ssCART-19 cells will not affect the IL-1α and IL-1β expression profiles.

Previous studies have shown that blocking IL-6/IL-6R does not affect the properties or therapeutic efficacy of CAR T cells [21]. Consistent with these observations, we found that ssCART-19 cells exhibited cell functions and anticancer activities similar to those of regular CART-19 cells in terms of response to tumor antigen stimulation, cell proliferation, and cytotoxicity to CD19+ cell lines and patient-derived primary malignant B cells. These data suggest that IL-6 knockdown in ssCART-19 cells may reduce the risk of CRS and CRES without sacrificing antitumor efficacy.

Last, we demonstrated that serum IL-6 levels were lower in a mouse xenograft model of leukemia after treatment with ssCART-19 cells than after treatment with regular CART-19 cells. The two types of CAR T cells showed similar antitumor toxicity, strengthening the hypothesis that IL-6 knockdown can improve the safety of ssCART-19 cell treatments without reducing antitumor effects in vivo.

The findings of our study are similar to those of a recent study that reported that GM-CSF knockout using CRISPR/Cas9 could reduce the sCRS rate and enhance T cell function in mice [25]. The shRNA-mediated gene knockdown, which silences endogenous genes at the RNA level, is one of the most common therapeutic approaches to treat genetic and acquired diseases [26]. One potential advantage of the shRNA gene knockdown technology approach compared to CRISPR/Cas9 gene knockout technology is that it avoids “off-target” genome editing effects at the DNA level that may cause unpredictable severe side effects, and complete abolishment of endogenous GM-CSF gene expression may also cause CAR T cells to fail to respond to physiological homeostasis needs. Given the incomplete understanding of the roles of each cytokine in the complicated human immune system and the potential “off-target” risks of CRISPR/Cas9 gene editing technology itself, the shRNA knockdown approach may be safer [27]. IL-6 knockdown also did not compromise T cell function, proliferation, differentiation or cytotoxicity.

In this study, we systematically evaluated the effects of IL-6 knockdown in ssCART-19 cells both in vitro and in vivo as a potential strategy to reduce the side effects of CAR T cell treatment. By knocking down IL-6 expression in ssCART-19 cells, the strength of the stimulation that triggers monocyte IL-6 release activity was reduced, which may further reduce the incidence of sCRS and improve safety profiles in the clinic. This approach may have major potential benefits for increasing patient survival, reducing the need for costly tocilizumab treatment, and decreasing the risk of neurological complications caused by CAR T cell therapy.

## Conclusion

The rapid elevation in CART-19-derived IL-6 expression induced by CD19-expressing cells may be one of the major triggering factors that enhances monocyte release of IL-6 and other proinflammatory cytokines, which may drive CRS development. Reducing the ssCART-19 cell-derived IL-6 expression level with an IL-6-specific shRNA could significantly reduce IL-6 release by monocytes, which could potentially decrease the severity or incidence of sCRS and related complications, without reducing ssCART-19 cell antitumor efficacy in vitro and in vivo. Thus, IL-6 knockdown in ssCART-19 cells may be a promising approach for improving safety in treating refractory and relapsed B lymphocyte leukemia and may also be used in other CAR T cells for improving clinical safety when treating other malignancies.

## Supplementary information

**Additional file 1: Figure S1.** CD19 expression in Raji and K562-CD19-LUC cells and CD14 expression in monocytes. **A** CD19 expression on Raji cells used for the mouse xenograft model and in vitro cytotoxicity assay. **B** CD14 expression in monocytes, as detected by flow cytometry.

**Additional file 2: Figure S2.** IL-6 knockdown in ssCART-19 cells have no effect on the IL-1 expression and secretion profile. IL-1α mRNA (**A**) and protein levels (B) in ssCART-19 cells and regular CART-19 cells after co-cultured with Raji. IL-1β mRNA (**C**) and protein levels (D) in ssCART-19 cells and regular CART-19 cells after co-cultured with Raji. Differences were assessed for significance using one-way ANOVA. Data are expressed as the mean + SD. NS, no significant difference, *p < 0.05.

**Additional file 3: Figure S3.** Effect of IL-6 knockdown on ssCART-19 cell differentiation and gene expression. **A** Differentiation of ssCART-19 cells and regular CART-19 cells, as detected by flow cytometry, Tscm (stem central memory T cells), Tcm (central memory T cells), Tem (effector memory T cells), Teff (effector T cells). **B** Top differentially expressed genes in ssCART-19 and regular CART-19 cells after coculture with Raji cells, as analyzed by RNA sequencing. **C** Gene ontology enrichment analysis of the RNA sequencing data showing the top 30 enriched functions.

## Data Availability

The data-sets used and/or analyzed during the current study are available from the corresponding author on reasonable request.

## Abbreviations

B-ALL: B cell acute lymphoblastic leukemia
CAR: Chimeric antigen receptor
CFSE: Carboxyfluorescein diacetate succinimidyl ester
GM-CSF: Granulocyte macrophage colony-stimulating factor
IFNγ: Interferon-γ
IL-6: Interleukin-6
IL-1: Interleukin-1
IL-2: Interleukin-2
NSG: NOD/SCID IL-2RγCnull
NC: Untransduced
PBMCs: Peripheral blood mononuclear cells
PBS: Phosphate-buffered saline
scFv: Single-chain variable fragment
TNF-α: Tumor necrosis factor-α
