# Supplementary figures and images for: Interleukin-6-knockdown of chimeric antigen receptor-modified T cells significantly reduces IL-6 release from monocytes
Source: Exp Hematol Oncol. 2020 Jun 8;9:11. doi: 10.1186/s40164-020-00166-2 (PMC7278071; doi:10.1186/s40164-020-00166-2)

A

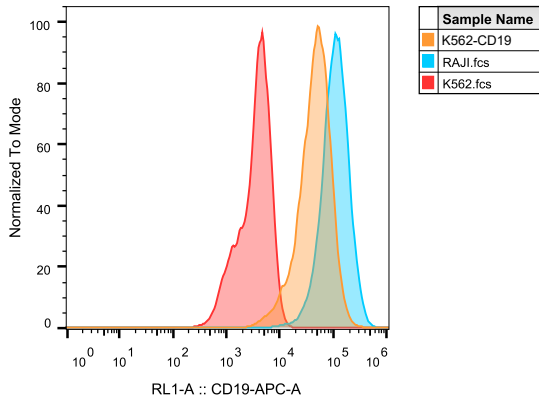

B

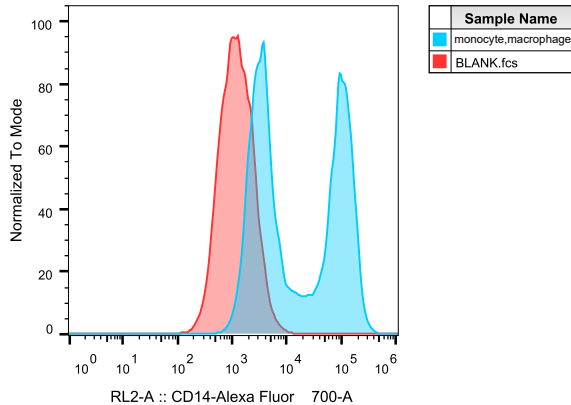

Supplement: Supplementary file 1 — Additional file 1: Figure S1. CD19 expression in Raji and K562-CD19-LUC cells and CD14 expression in monocytes. A CD19 expression on Raji cells used for the mouse xenograft model and in vitro cytotoxicity assay. B CD14 expression in monocytes, as detected by flow cytometry. [file 40164_2020_166_MOESM1_ESM.pdf]

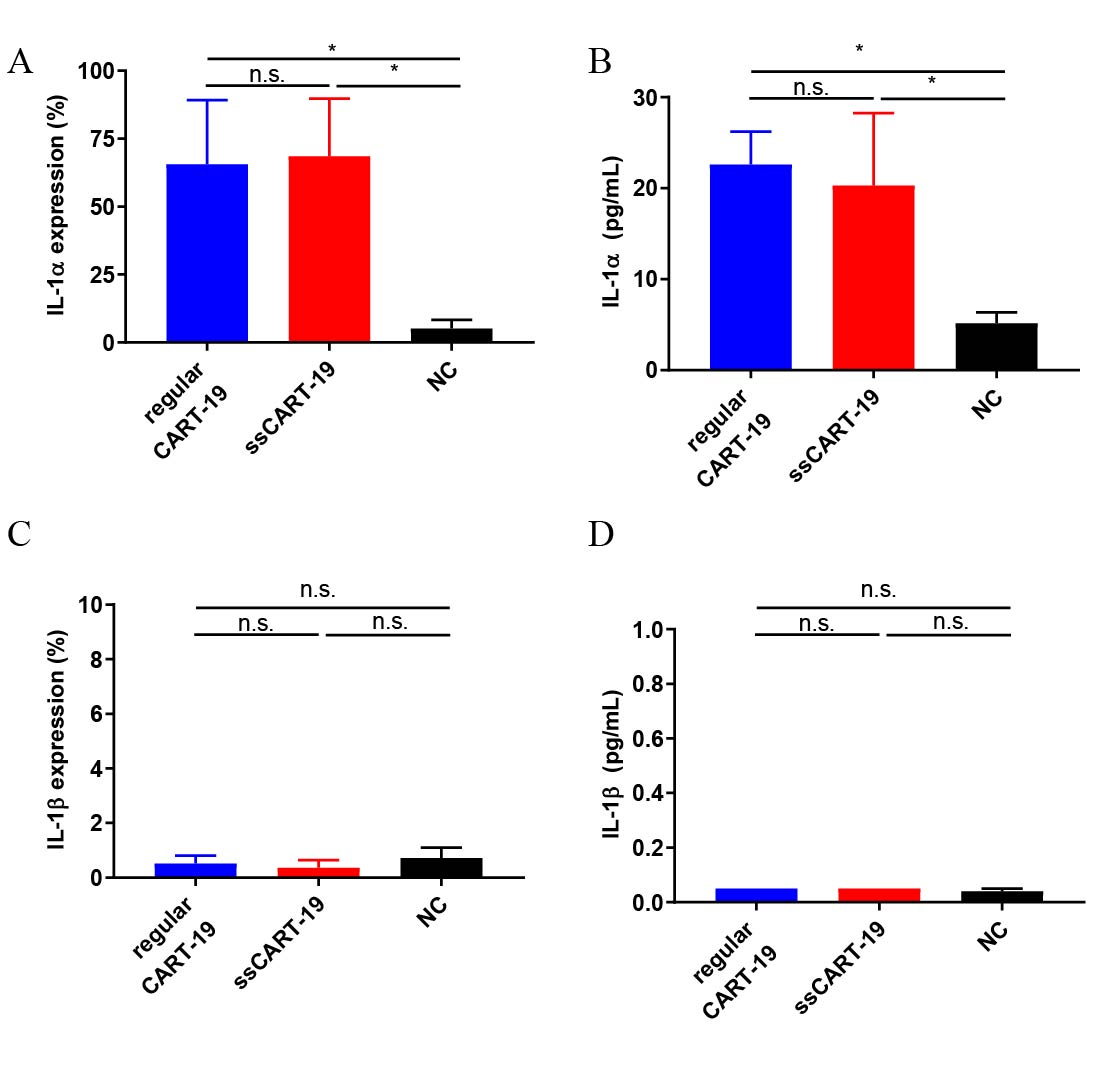

Supplement: Supplementary file 2 — Additional file 2: Figure S2. IL-6 knockdown in ssCART-19 cells have no effect on the IL-1 expression and secretion profile. IL-1α mRNA (A) and protein levels (B) in ssCART-19 cells and regular CART-19 cells after co-cultured with Raji. IL-1β mRNA (C) and protein levels (D) in ssCART-19 cells and regular CART-19 cells after co-cultured with Raji. Differences were assessed for significance using one-way ANOVA. Data are expressed as the mean + SD. NS, no significant difference, *p < 0.05. [file 40164_2020_166_MOESM2_ESM.jpg]

**A****Tcm/CD8**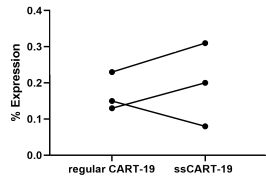**Teff/CD8**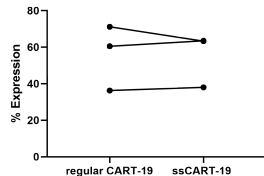**Tscm/CD8**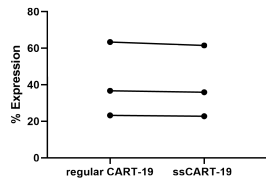**Tem/CD8**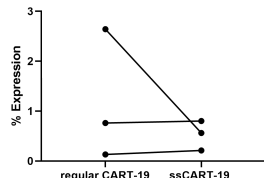**B**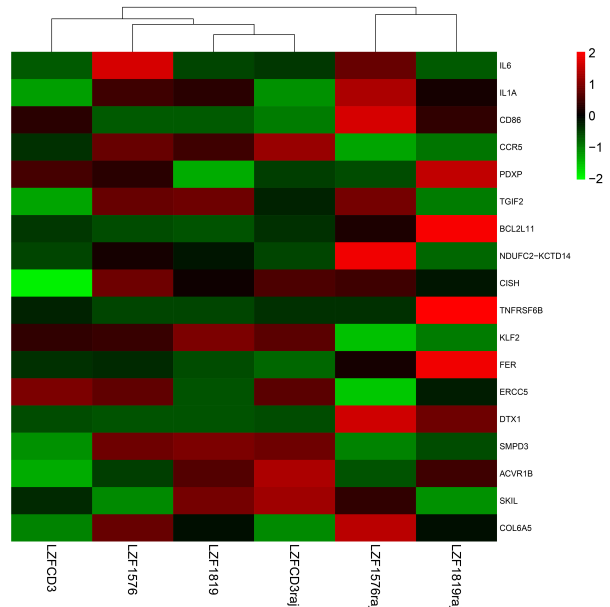**C****Top 30 of GO Enrichment**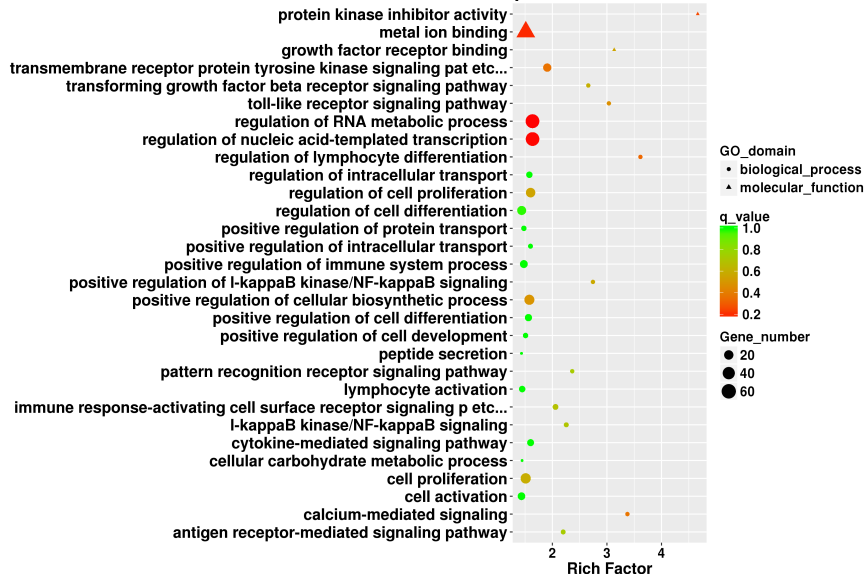

Supplement: Supplementary file 3 — Additional file 3: Figure S3. Effect of IL-6 knockdown on ssCART-19 cell differentiation and gene expression. A Differentiation of ssCART-19 cells and regular CART-19 cells, as detected by flow cytometry, Tscm (stem central memory T cells), Tcm (central memory T cells), Tem (effector memory T cells), Teff (effector T cells). B Top differentially expressed genes in ssCART-19 and regular CART-19 cells after coculture with Raji cells, as analyzed by RNA sequencing. C Gene ontology enrichment analysis of the RNA sequencing data showing the top 30 enriched functions. [file 40164_2020_166_MOESM3_ESM.pdf]
